# Analysis of Thermomechanical Properties and the Influence of Machining Process on the Surface Structure of Composites Manufactured from Metal Chips with a Polymer Matrix

**DOI:** 10.3390/polym14173501

**Published:** 2022-08-26

**Authors:** Adam Gnatowski, Rafał Gołębski, Jana Petru, Marek Pagac

**Affiliations:** 1 Department of Technology and Automation, Czestochowa University of Technology, 42-200 Czestochowa, Poland; 2 Department of Machining, Assembly and Engineering Metrology, Technical University of Ostrava, 70800 Ostrava, Czech Republic

**Keywords:** polymer composites, metallic filler, machining, DMTA testing, roughness, surface integrity parameters

## Abstract

Nowadays, the dynamic development of the entire market of composite materials is noticeable, which is very often associated with the need to use waste or recycled materials in their production. In the process of producing composites themselves, the easy possibility of shaping their mechanical and thermomechanical properties becomes apparent, which can be a big problem for materials with a homogeneous structure. For the tests, samples made of a combination of acrylic–phenolic resin with fine aluminum and brass chips were used. The tests were performed for composite samples produced by pressing. This paper presents the results of the DMTA method of the conservative modulus and the tangent of mechanical loss angle of the composite, a detailed stereometric analysis of the surface after machining, roughness parameters and volumetric functional parameters were performed. For the tested samples, changes in the values of the conservative modulus and the mechanical loss coefficient were recorded, which indicated significant differences for the composite with brass chips in relation to composites with aluminum chips. In the case of the composite with aluminum chips, slight changes in the conservative modulus were recorded in the glass transition phase and the elastic deformation phase at different frequencies. In contrast, for composites with brass, slight changes were recorded in the entire range of the course of the conservative module as a function of temperature when different excitation frequencies were applied. In relation to the polymer matrix, a significant increase in the value of the conservative modulus of composites was recorded in the entire temperature range of the test. Significant differences were recorded in the study of the surface of composites in the case of using different materials obtained after machining as fillers. The dependences of the amplitude parameters of the surface after machining the sample made of phenolic–acrylic resin prove the poor performance properties of the surface. The use of chips in the composite significantly changed the surface geometry.

## 1. Introduction

Polymer-based composites reinforced with metallic particles are increasingly used in modern constructions, displacing traditional materials in various industries. The matrix of composite materials can be metals, ceramics and plastics. The function of the matrix is to keep the reinforcing phase in a specific place in the material structure and to counteract deformation under the influence of loads, transferring the stresses to the components of the reinforcing phase. The selection of an appropriate metal filler for polymer modification causes changes in its specific mechanical [1] and thermal [2] properties or a reduction in the price of a given product with similar properties [3]. In recent years, there has been a steady increase in material recycling, which consists of the processing of unsuitable elements into raw materials that could be reused in the production of new end products with similar mechanical properties as polymeric materials with high thermomechanical parameters [4]. Materials from machining processes, i.e., post-production metal chips, which have not been processed so far, are also fields of interest [5]. Depending on the selected type of metal reinforcement, we can theoretically predict the properties of a given composite. Metal–polymer composites combine favorable properties such as electrical and thermal conductivity with a lower density than pure metal [6]. In the case of using aluminum powder as a filler for the polymer in the tests presented in [7], an increase in the strength parameters of the tested materials was obtained. The work [8] presents the results of research and analysis of the electrical, mechanical and thermal properties of polyvinyl chloride (PVC) composites filled with various contents of aluminum powder in the range from 0 to 40% by weight, indicating the favorable properties of the produced composite, including increasing the thermal stability of composite samples with an increase in the content of aluminum filler. Sehajpal et al. [9] presented tests of poly (methyl methacrylate) with a filler containing silver, aluminum and copper particles, increasing thermal conductivity and strength parameters, respectively. Bhagyashekar et al. [10] carried out tests on a composite consisting of metallic and non-metallic fillers. The results of tests carried out on composites containing three different fillers, in the form of particles, metallic materials (Cu and Al), ceramic (SiC) and grease (Gr), showed that the properties changed with increasing filler content. The hardness of the composites increased with the increase in the filler content, except for the composites with a Gr filling, which showed the opposite tendency. The works of many other authors take into account both the issues related to the production method and theoretical descriptions of the modification of the polymer material with fillers. In the work of Akhtar et al. [11], hybrid fillers—alumina graphene (Gr-Al_2_O_3_)—were synthesized and added to the epoxy matrix in order to improve the thermal properties of the composite. Abdulkareem et al. [12] presented the effect of the content of metal filler and particle size on the hardness and tensile strength of polystyrene composites. In the work [13], polypropylene (PP) composites filled with aluminum with various shapes of filler particles and contents ranging from 0% to 55% by volume were investigated. The influence of loads and the shape of the filler particles on the properties of the composites was identified. Nurazreena et al. [14] investigated the electrical properties and tensile strength of composites formed by adhibition metal powders such as aluminum (Al), copper (Cu) and iron (Fe) into a high-density polyethylene (HDPE) matrix. Similar studies were conducted by Lotfy et al. [15] and Tavman et al. [16]. The work [17] concerns the preparation and characterization of composite materials produced by compression molding of a mixture of aluminum flakes and nylon 6 powder. Electrical conductivity, density, hardness and morphology of the composites were investigated, obtaining favorable properties of the composites. Other studies with an aluminum filler were conducted by Schricker et al. [18]. The joining zone of semi-crystalline polyamide 6 with aluminum was investigated in the laser joining process and the mechanical properties of the joint were assessed. The mechanical properties were tested up to cohesive failure. Furthermore, the mechanical properties were correlated with the results of hardness, morphology, differential scanning calorimetry (DSC) and X-ray diffraction (XRD) results. The properties of electro-contact-sintered metal–polymer composite materials were analyzed in the work of Kovtun et al. [19]. Bloor et al. [20] investigated metal–polymer composites containing a filler dispersed in an insulating polymer matrix, prepared by mixing components at high speed. Dasture and Kelkar [21] investigated the mechanical, structural and morphological properties of a low-density polyethylene (LDPE) composite with an aluminum filler. In contrast, research on the use of metal–polymer composites with a metal filler in the form of brass was carried out by Eddoumy et al. [22]. To analyze the benefits of adding brass, the friction and wear were investigated, as well as the thermomechanical properties of the brass-filled material. The paper [23] presents an alternative method to the classical melting process, resembling the powder metallurgy process, in the recycling of metal chips mass-produced as a result of machining. The influence of the degree of reinforcement on the mechanical properties and microstructure of the composite material was investigated and the obtained composite materials were compared with the industrial brass alloy. Adding a filler or forming a polymer blend reduces the cost of producing a given product. Using known manufacturing techniques, various composites can be produced depending on the content and type of filler used. The modification of polymers significantly influences the treatment process [24].

The subtractive processing of composite materials is of great importance in the production process of components that are subject to high quality requirements, with particular emphasis on dimensional tolerances. Due to the random structure of the material, machinability tests of composites using various tools and machining strategies are increasingly being carried out. This was pointed out by Usca et al. [25] by conducting this type of research and determining the optimal composition of the composite in order to improve the machinability factors in the applied machining process. Very often, the produced composite materials cooperate with each other, undergoing tribological wear [26]; therefore, for a better quality of components, the parameters of the finishing process are of great importance, and the very assessment of tribological characteristics in the testing processes of composites is increasingly important. Conventional machining processes, such as turning, drilling or milling, can be applied to composite materials, provided that the appropriate tool design, working conditions and cutting parameters are adopted. Due to the anisotropic and heterogeneous structure of composites, the processing of composites becomes more and more demanding. The processing of heterogeneous materials (of different hardness) causes their uneven cutting—the matrix material behaves differently during processing—or filling [27]. Depending on the adopted cutting parameters and the composite structure, the energy consumption of the process may vary considerably. An article by Usca et al. [28] proposed a very accurate comprehensive approach to assess the energy consumption of the process in the context of the machinability criteria of the tested composites. Metal matrix composites pose a much greater challenge to the processing process compared to composites with a metal filler. The heterogenic structure of such material very often leads to damage to the cutting tool during machining, which is a consequence of the presence of relatively harder particles in the material [29], which consequently leads to an increase in surface roughness and loss of stability of the functional parameters of the surface after machining. Therefore, in this work it seems justified to undertake a research task to evaluate the produced composite with a metallic filler.

This paper presents research on the production of a polymer matrix composite with metallic fillers. In quantitative terms, the aim of the study was to determine the scope of the impact of modification by adding a filler to the material on the quality parameters of the processed elements, samples produced from composites based on metal chips. This work will also include an analysis of the impact of modifying the thermomechanical properties of the polymer matrix through the use of a filler on the improvement of the machinability of the polymer material, and, consequently, the qualitative assessment of the improvement of the surface layer condition, taking into account several parameters such as: surface roughness, volumetric functional parameters and analysis of surface stereometry.

## 2. Materials and Methods

**Figure 1:**
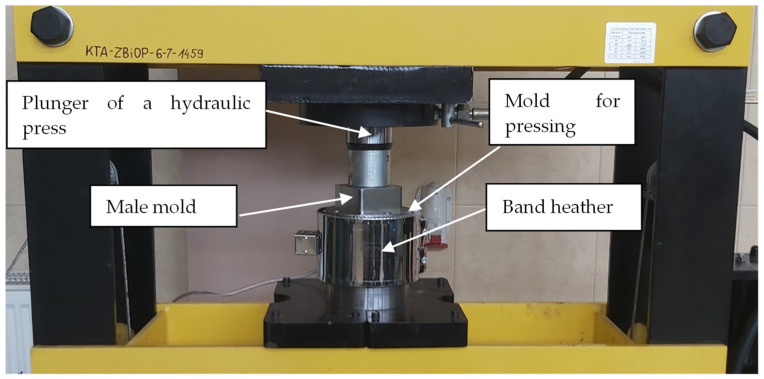
Stand for manufacturing composite samples.

The tests were carried out for samples made of a combination of Dialok 939P acrylic–phenolic resin (Bitrez Ltd, Bradley Lane, Standish, UK) with fine aluminum (2017A aluminum) and brass (Brass CuZn37Pb0.5) chips with a fraction of 0.6–0.75 mm in the amount of 95% by weight. A hydraulic press (Viber-System, Gorzow Wielkopolski, Poland) with a pressing force of 75 tons was used to prepare the samples. The 80 × 80 × 25 mm samples were formed in the press mold under a compaction pressure of 61.3 MPa. The plasticization temperature was 95 °C and the cross-linking temperature was 180 °C, with a time of 15 min. Figure 1 shows a stand for manufacturing samples by pressing. The mold with a diameter of 110 mm was heated to the nominal operating temperature using a 2.4 kW band heater. 

**Figure 2:**
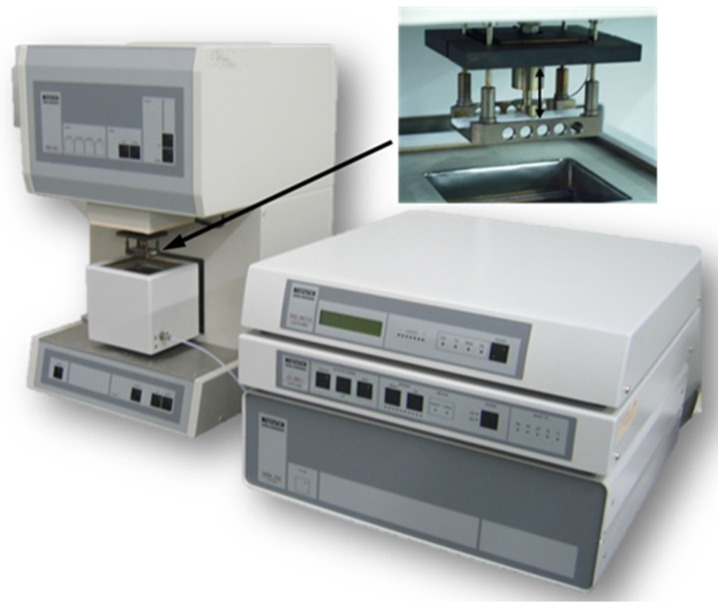
Research stand Netsch DMA 242 C.

In order to compare the test results of composites to the polymer matrix, samples were also made of pure resin; in the production process, the same processing parameters were used as in the samples with the addition of fillers. Thermal analysis of dynamic mechanical properties—DMTA—was carried out in accordance with the standard [30] with the NETSCH DMA 242 C device (Netzsch Group, Selb, Germany) at a temperature of 70 to 300 °C, heating at a rate of 2 °C/min and at frequencies of 1 Hz and 10 Hz. The dimensions of the samples were 50 × 10 × 4 mm. Based on the values of force and deformation (read by measuring sensors—Netzsch Proven Excellence, Selb, Germany), taking into account the dimensions of the sample, the value of the conservative modulus E’ and the tangent of the mechanical loss angle tgδ were calculated [31,32]. The results are presented in the form of a graph of changes in the conservative modulus E’ and the tangent of the mechanical loss angle tgδ as a function of temperature. Figure 2 shows the DMTA test stand with a three-point bending holder placed without fixing the sample. The dynamic mechanical–thermal analysis used in the research is one of the methods that allow the estimation of the changes occurring in the material during bending in a wide range of temperature and frequency of load changes. The knowledge of the course of these changes allows the establishment of the relationship between the molecular parameters and the mechanical properties of materials [32].

**Figure 3:**
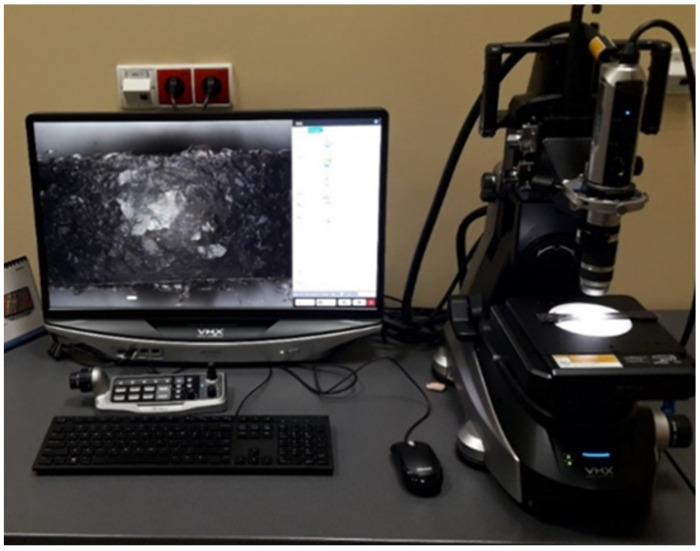
Keyence VHX 7000 confocal microscope.

Macroscopic (visual) examination of the surface was carried out using a Keyence VHX 7000 (Keyence Ltd, Milton Keynes, U.K) confocal microscope (see Figure 3), which uses white light and laser light. It scans the surface of a given material, collecting information about the roughness and shape of the surface, and creates an optical image. The measurement process takes place without contact with an accuracy of nanometers. The high imaging resolution allowed for the precise measurement of surface quality and analysis in terms of measuring defects, microcracks and porosity. The study of the surface macroscopic structure of composites using a Keyence VHX 7000 digital microscope (Keyence Ltd, Milton Keynes, UK) was carried out on samples of acrylic–phenolic resin, acrylic–phenolic resin with aluminum chip filler and brass filler. The surfaces of the samples, produced by pressing after the machining process, were observed. All tests were preceded by device calibration in order to improve the quality of results. The use of macroscopic imaging made it possible to appreciate the uniformity of the filling distribution on the machined surface.

### Composite Machining Process

**Figure 4:**
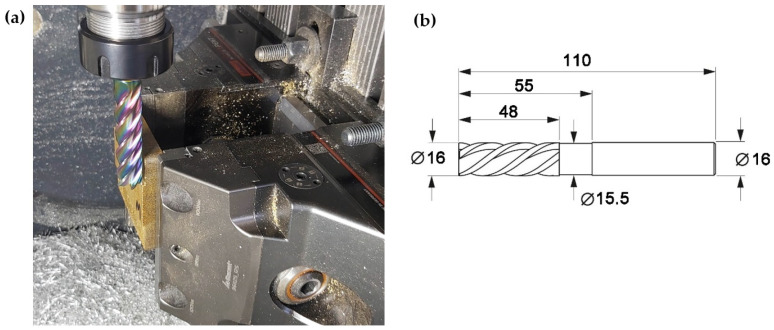
Composite processing, (**a**) sample mounting, (**b**) parameters of the tool used in the process.

The samples produced by pressing were subjected to machining in order to assess their machinability and the condition of the surface after processing. Machining materials with a heterogeneous structure is a big challenge for machining. The main difficulties when processing composite materials are unsatisfactory surface quality and difficulties related to the correct selection of tools and parameters. During machining, it is possible to damage the surfaces, as issues typical for the machining of heterogeneous materials exist that are not present when machining metal and non-metal materials. The processing was performed on a DMG MORI CMX50U (DMG MORI, Famot Pleszew, Poland) numerically controlled milling machine. The machined materials, composite blank plates, were fixed in a vice dedicated to multi-axis machining using claw jaws—see Figure 4a). When mounting, particular attention was paid to the clamping force of the jaws, which did not exceed 20 kN, in order to reduce the occurrence of internal stresses in the material during processing. A solid carbide milling cutter (GARANT—Hoffman Group, Munich Germany) with a diameter of 16 mm with unequal spacing helix angle 50 deg (Figure 4b) with five blades was used for machining. The tool [33] with DLC (diamond like carbon) coating is used for machining brass, aluminum and also polymer materials (materials giving short or long chipping during machining). Processing parameters were adopted: feed rate per tooth fz = 0.05 mm/tooth, cutting speed Vc = 550 m/min, cutting depth ap = 25 mm, cutting contact width ae = 1 mm.

Tool shank according to DIN 6535 HA with h5 tolerance. A tool holder was used, made in accordance with ISO 7388-1, type ER32 SK40 A100, maintaining a rotational accuracy of ≤3 µm and balancing accuracy of G 2.5 at a rotational speed of 25,000 min^−1^.

## 3. Test Results and Analysis

**Figure 5:**
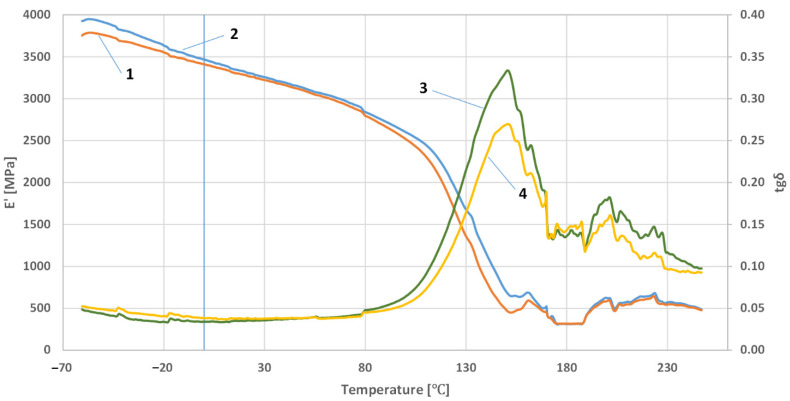
The dependence of the conservative modulus and the tangent of the mechanical loss angle on the resin temperature: at a frequency of 1 Hz—1, 3; at a frequency of 10Hz—2, 4.

**Figure 6:**
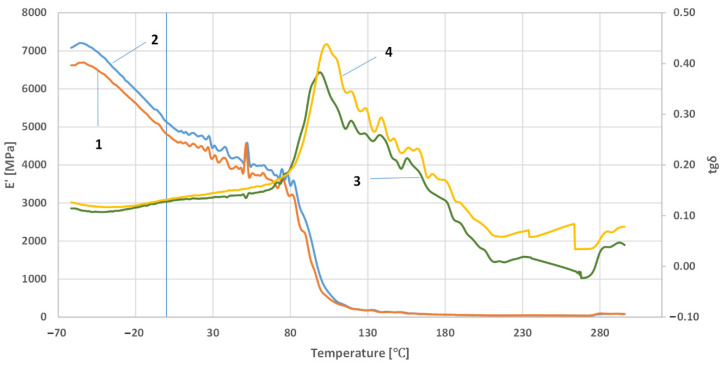
The dependence of the conservative modulus and the tangent of the mechanical loss angle on the temperature of the composite of resin with aluminum chips: at a frequency of 1 Hz—1, 3; at a frequency of 10 Hz—2, 4.

**Figure 7:**
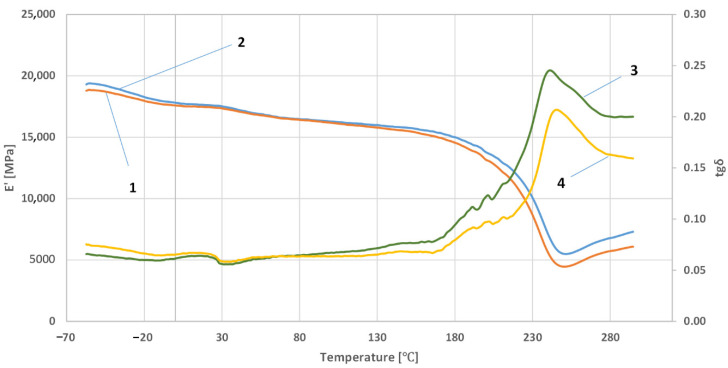
The dependence of the conservative modulus and the tangent of the mechanical loss angle on the temperature of the resin composite with brass chips: at a frequency of 1 Hz—1, 3; at a frequency of 10 Hz—2, 4.

Figure 5, Figure 6 and Figure 7 show the results of the research on the dependence of the conservative modulus and the tangent of the mechanical loss angle on the temperature of the acrylic–phenolic resin and the composite of acrylic–phenolic resin with aluminum chips and acrylic–phenolic resin with brass chips.

**Figure 8:**
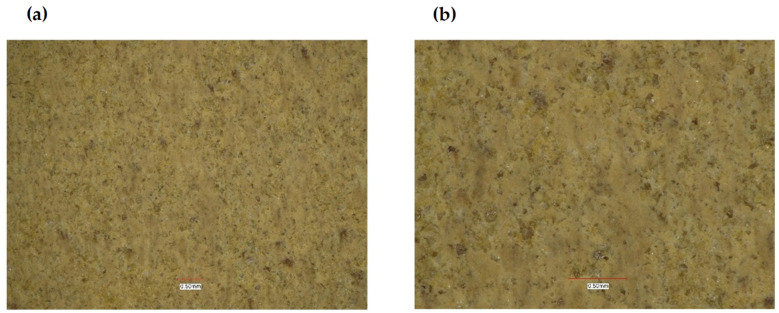
Acrylic–phenolic resin: (**a**) magnification 50×, (**b**) magnification 100×.

**Figure 9:**
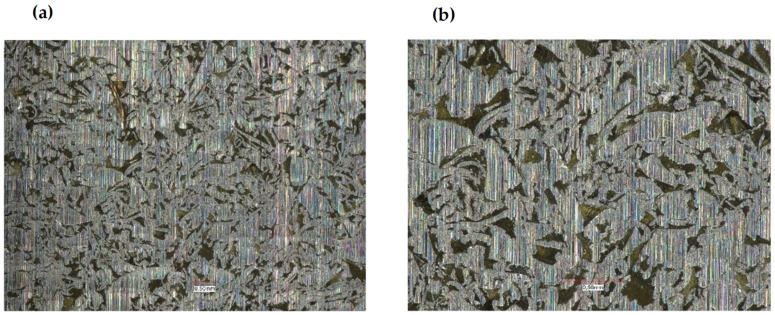
Composite of acrylic–phenolic resin with aluminum chips: (**a**) magnification 50×, (**b**) magnification 100×.

**Figure 10:**
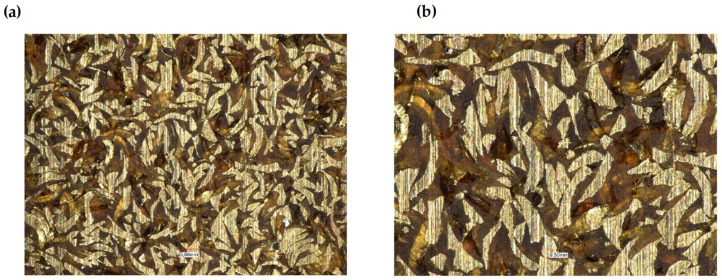
Composite of acrylic–phenolic resin with brass chips: (**a**) magnification 50×, (**b**) magnification 100×.

The presented research shows that adding aluminum or brass chips as a filler allows the obtainment of composites with satisfactory thermomechanical properties. For the tested samples, it was noted that the values of the conservative modulus of the filled materials increased. In the case of composites with brass filler, significant changes were recorded in the entire range of the curve. The analysis of the recorded values of the conservative modulus and the tangent of the mechanical loss angle shows significant differences for the composite with brass chips compared to the composites with aluminum chips. In the case of the composite with aluminum chips, changes in the conservative modulus were recorded in the glass transition phase and the elastic deformation phase at different frequencies. In contrast, for composites with brass, changes were recorded in the entire range of the course of the conservative modulus as a function of temperature with the use of different frequencies. For the sample with aluminum chips and the addition of 5% resin in the glass transition phase, the values of the conservative modulus were lower by approx. 10,000 ÷ 12,000 MPa compared to the composite with brass chips. In the field of high-elastic deformations, an increase in the value of the conservative modulus for composites with brass chips was recorded similarly, while it was about half lower than in the glass transition phase, whereas the high-elastic deformation phase shifted towards higher temperature values. Changes in the value of the tangent of the mechanical loss angle and its maximum may indicate the stiffness of the material and properties such as hardness and toughness, which affect the machining process. The maximum value of the tangent of the mechanical loss angle was recorded for the acrylic–phenolic resin at the temperature of 151.5 °C at the frequency of 1 Hz, and for the material with the addition of aluminum chips, for 108 °C, an increase in the maximum value was recorded. In the case of acrylic–phenolic resin with the addition of brass chips, the maximum value of the tangent of the mechanical loss angle was shifted towards a much higher temperature of 241.5 °C. The macroscopic image of the treated surfaces of the test specimens made on the Keyence microscope at 50× and 100× magnification is shown in Figure 8, Figure 9 and Figure 10.

The surface of the sample made of resin is characterized by an uneven, rough surface, and it contains many pointed protrusions and steep depressions. Such an image of the surface results from the structure of cross-linked acrylic–phenolic resin. The processing of this material causes it to crumble, which creates a large number of small cavities with sharp edges. The lack of material capable of carrying loads and dissipating them results in the tendency of more strongly integrated fragments to detach from the rest of the material on the contact lines with weaker cross-linking. Samples made of a composite of resin with aluminum chips and a composite with brass chips are characterized by an uneven, rough surface containing many smoothly ending protrusions and depressions. A significant relationship was recorded between the type of filler and the unevenness of the surface. The characteristics of the composite material indicate a certain flakiness in the structure of the surface. Such an image of the surface results from the structure of the polymer and the filler used. The use of brass in this composite significantly changed the geometry of the surface. The surface visible on the base resin sample changed to the more formed side after the machining process. The filler in the polymer matrix transfers the loads deeper into the composite, causing them to disperse. However, the use of a filler significantly influenced the smoothness of the surface. In the case of composites with a filler in the form of aluminum chips, the convexities on the plane do not occur evenly over the entire surface, but only in a certain area. Such behavior of the composite results from the presence of such a type of filler in these places, which means that the crack line, possibly formed during the loads, may run in these places. In terms of the homogeneity of the surface, a high regularity was observed when using the filler in the form of brass chips.

In general, both natural-untreated and manufactured surfaces have the original shape or form with varying degrees of structure, waviness and roughness, considering them both as 2D as well as 3D. All surface features will contain both controlled and uncontrolled characteristics. If the goal is to determine the surface three-dimensional texture of a surface, the results can also be rigorously linked to the corresponding parameters defined in the increasingly widespread ISO 25178 standard [34]. In the production of industrial machining, the analysis of data related to generally accepted standards may make the obtained results identify more of the technological problems of the process itself. In the next stage of the research, the processed samples—(a) 100% resin, (b) 5% resin, 95% brass, (c) 5% resin, 95% aluminum—were analyzed on the treated surfaces on a laboratory contact profilographometer Taylor Hobson, Talysurf 120. A measuring blade with a 2 µm tip was used for the measurement. The measurement area was determined randomly in the range of 9 × 7 mm, the resolution of the measuring blade’s pass was assumed every 5 µm in the machining direction (tool blade operation). Measurement time for 1 sample was 7 h 30 min, and it did not include the development of functional and amplitude data of the surface profile. The roughness of the treated surfaces was measured in the direction parallel to the machining direction. Table 1 presents the basic parameters of the roughness profile amplitude: The Ra parameter, which reacts poorly, has local changes in the surface structure, and therefore its value often does not give a clear picture of the surface condition, taking into account local changes; therefore, the roughness parameter Rz was additionally selected to describe the surface condition. The analysis of the Rp parameter, the height of the highest elevation of the profile, and Rv of the depth of the lowest cavity of the roughness profile was performed. The roughness profile skewness coefficient Rsk was also determined—commonly referred to as the profile asymmetry coefficient—along with the coefficient Rku—surface kurtosis (flattening)—which gives an image of the width of the roughness profile grooves. For the roughness height assessment parameters, the following were analyzed: Sq—mean square deviation of the surface unevenness height from the reference plane, Sp—height of the highest elevation of the surface, Sv—depth of the lowest cavity, Sz—size of the roughness profile, mean arithmetic height of the surface in relation to the 10 largest elevations of the profile and Sa—arithmetic mean deviation of the surface from the mean surface, which is the arithmetic mean of the absolute values of the deviations of the height of the surface from the mean surface. Very important functional parameters of the surface are the material fraction functions, which represent the dependence of the surface material fraction on its height. We can interpret that it is a 2D to 3D transformation of the analysis and, similarly to the profile, it can be interpreted as a cumulative probability function of the coordinates Z (x, y) [35].

From this curve—also like in the 2D analysis—numerical parameters are calculated and subsequent functions are derived. They include: the relative material area factor Rmr, which is the percentage of material at a given height; Smq, which is the relative material fraction at the intersection of flats and depressions; Svq, which is the mean squared deviation of the valleys as the slope of the regression line through the valley region; and Spq, which is the difference in levels between the beginning and end of the valley region.

**Figure 11:**
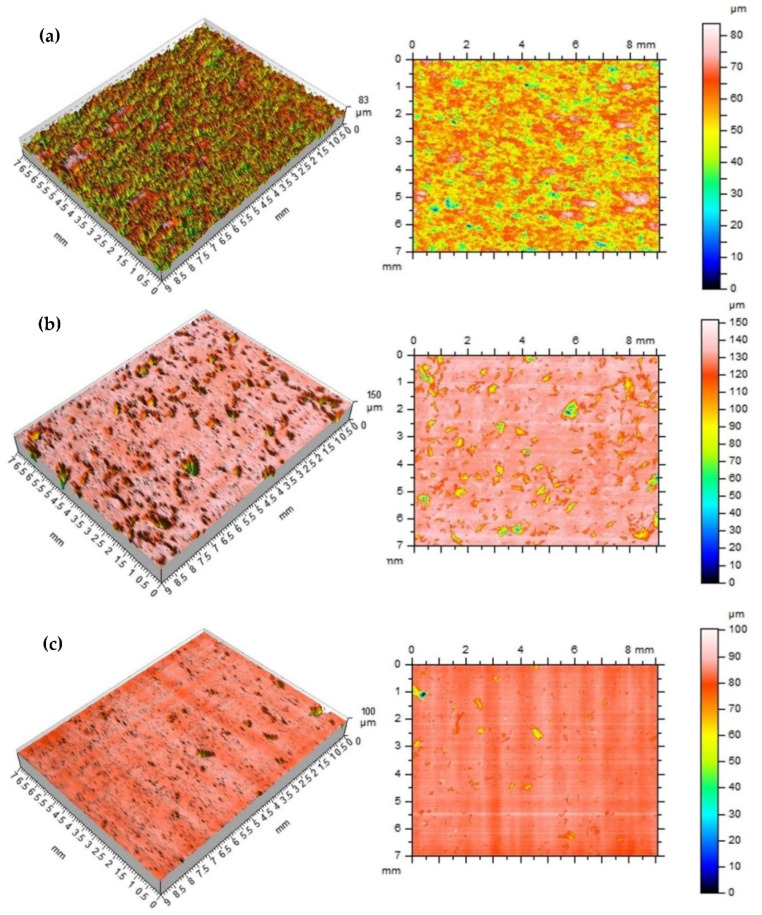
The stereometric distribution of the machined surface—sample (**a**), 100% resin; sample (**b**), 5% resin and 95% brass; sample (**c**), 5% resin and 95% aluminum.

The microgeometric structure of the surface layer was also assessed; the measurement was made in the central section of the cut area perpendicular to the machining direction. Figure 11 shows the original structure of the stereometric distribution of unevenness of the processed composite samples.

**Figure 12:**
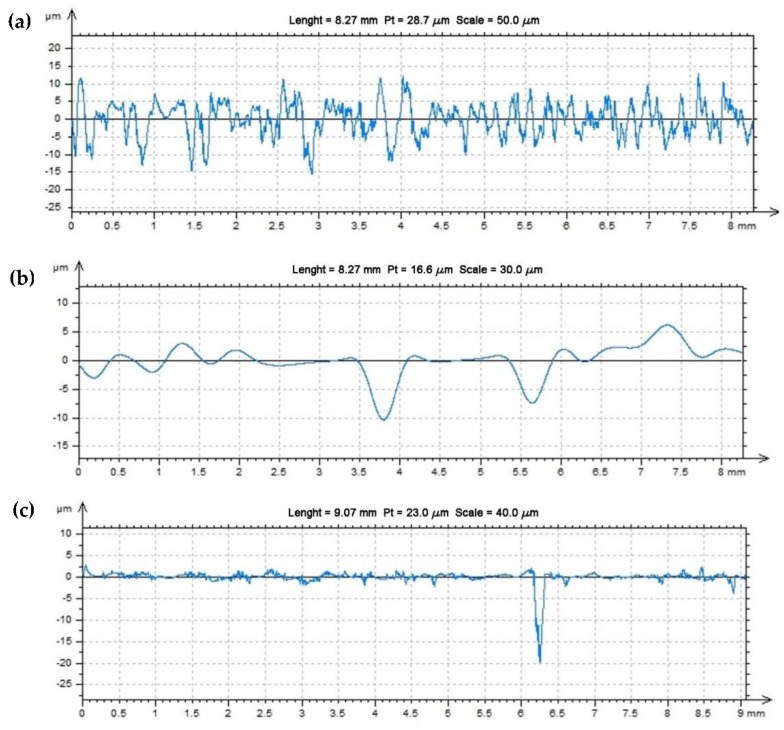
The roughness Ra profile parameter—sample (**a**), 100% resin; sample (**b**), 5% resin and 95% brass; sample (**c**), 5% resin and 95% aluminum.

For two composites and an acrylic–phenolic resin, the roughness of the machined surfaces was measured in the direction of the tool feed, and also on a laboratory contact profilographometer by Taylor Hobson (Taylor Hobson, New Star Road, UK, 2012). Figure 12 shows the roughness profile distribution for the Ra parameter. The measurement length was limited to 9 mm.

**Figure 13:**
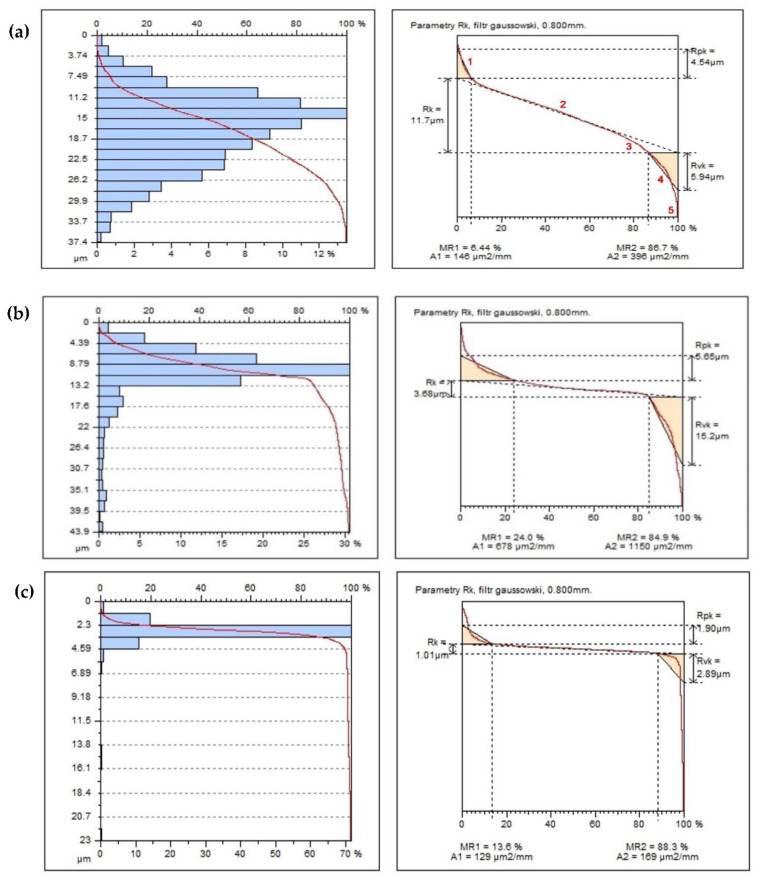
Material ratio in function of the profile height—(**a**) 100% resin, (**b**) 5% resin and 95% brass, (**c**) 5% resin and 95% aluminum.

The main profile parameters are defined in the ISO 4287 standard [36]. The measured profile is a series of height values in function of the lateral position. Heights are references by convention from the mean line, which is calculated as the mean value of all heights. Figure 13 shows the surface functional parameter Rmr as the ratio of the material at a given depth. This parameter gives the percentage of material cut at a given depth from the top of the profile. The material fraction surface evaluation method uses the material fraction distributor, i.e., a representation in which the material fraction is expressed as a probability on the normal distribution grid. The material share is expressed in multiples of the standard deviation, deposited evenly on the horizontal axis (Figure 13). The curve is divided into 5 areas depending on the zones of elevation formation [35]: inequalities: zone 1—high peaks (or unexpected impurities), 2—flattening area-stable, 3—unstable transition area (transition from the flattened zone to the valley zone), 4—dimple area and 5—deep scratches and material defects, as seen indicated as sample in Figure 4a. Characterizing parameters are calculated for the zones divided in this way, as seen in Table 1: material surface ratio Smr, which is the percentage of material for a given height. Smq is the relative material fraction at the intersection of flats and depressions. Svq is the mean squared deviation of the valleys as the slope of the regression line through the valley region and Spq is the difference in levels between the beginning and end of the valley region.

Main profile parameters are defined in the ISO 4287 standard [36]. The measured profile is a series of height values in function of the lateral position. Heights are references by convention from the mean line which is calculated as the mean value al heights. Figure 13 shows the surface functional parameter as the ratio of the material at a given depth. This parameter gives the percentage of material cut at a given depth from the top of the profile. The reference may also be taken from the center line or another reference height. The bearing area curve allows the description of the diversity of the properties of the tested profile that change with its depth. The bearing area curve (BAC), or Abott–Fireston curve, determines, for a given depth (vertical axis of the graph), the percentage of material cut in relation to the coated material. The following parameters were obtained: the peak volume of the surface material Vmp, the core volume of the surface material Vmc, the volume of the hollow core surface space Vvc and cavity volume of surface Vvv.

**Figure 14:**
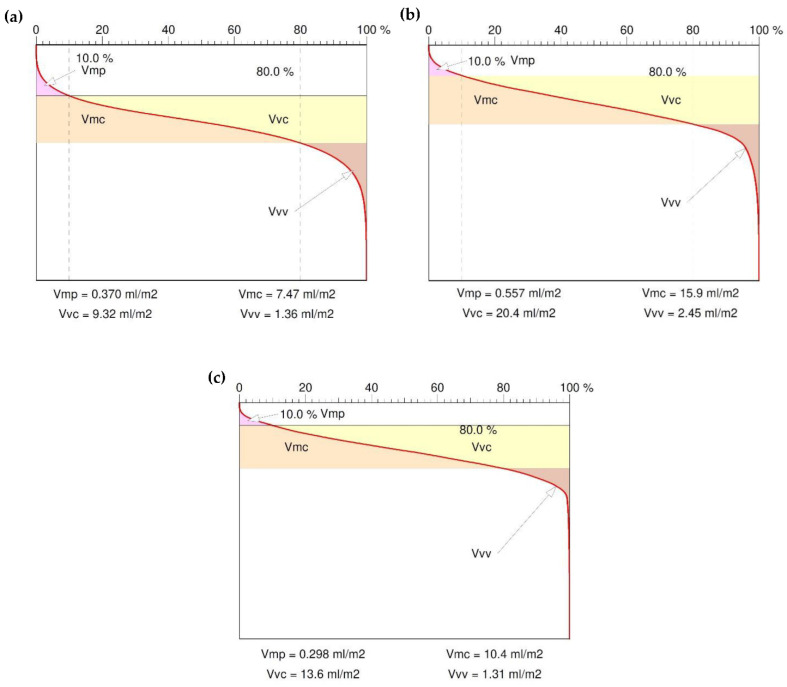
Abbott–Firestone curve (bearing area curve), surface volumetric functional parameters—(**a**) 100% resin, (**b**) 5% resin and 95% brass, (**c**) 5% resin and 95% aluminum.

The Vmp parameter determines the compliance of the surface during sliding and rolling lapping. In this case, the small value of this parameter indicates high abrasion resistance, i.e., good running-in behavior. In contrast, the void volume of a recess in the surface Vvv is a measure of the oil holding capacity of the surface—Figure 14. Based on the above volumetric parameters, we can forecast the surface exploitation process.

## 4. Conclusions

The addition of machining waste in the form of metal chips as a filler to the acrylic–phenolic resin made it possible to obtain composites with good thermomechanical properties and machinability. In the case of the analyzed composites, there was an increase in the value of the conservative modulus, differences in the maximum tangent temperature of the mechanical loss angle and significant changes for the samples with the filler in the form of brass chips. Taking into account the analysis of the obtained test results using the DMTA method, it can be concluded that the highest parameters were obtained for the acrylic–phenolic resin composite with the addition of brass chips. The composite was characterized by good stiffness and the ability to dampen vibrations while maintaining high values of the conservative modulus in the entire test temperature range.

The analysis of the surface structure of the composites indicated the uniformity of chip distribution for both aluminum and brass resin composites. For a composite with aluminum chips, the size of the visible elements of the surface structure is characteristically reduced, whereas their density increases for composites with brass chips. The research revealed a significant effect of the type of filler on the properties of the produced composite based on recycled materials. The use of chips in the composite significantly changed the surface geometry. The visible area on the resin base sample changed to more wavy level changes. The resin-capped chips transferred the loads into the composite, causing them to dissipate. In addition, the geometry of the chip, which was in the form of a flat polygon, caused the force acting perpendicular to its plane to be transferred to the resins through a larger surface, which reduced its chipping.

The treatment of the homogeneous resin caused the surface layer to crumble, which created a large number of small defects with sharp edges. The original profile of the roughness parameter Ra showed high amplitude tendencies and the kurtosis parameter Rku (flattening) of the surface, giving the image of the width of the grooves of the roughness profile, was very low; as a consequence, Rmr, the material fraction index (the amount of material in the core of the surface layer), was also low. Such dependences of the amplitude parameters of the surface after machining the sample from acrylic–phenolic resin proves the poor functional properties of the surface. 

The void volume of a recess in the surface Vvv is a measure of the oil holding capacity of the surface, and both the brass and aluminum specimens of composites had the correct ability to keep the lubricant on the surface.

In the machining process of composites, brass showed a tendency to chipping on the surface. The composite with aluminum filler had the highest surface smoothness; in addition, the amplitude roughness and functional parameters of the surface, with particular attention to the material ratio, were very satisfactory. The volumetric parameters of the surface also prove the correct proportion of the presence of the material filling in the core of the surface layer.

## Figures and Tables

**Table 1:** Measurement results, surface roughness and functional parameters, measured perpendicular to machining direction.

Surface Parameters	Sample (a) 100% Resin	Sample (b) 5% Resin 95% Brass	Sample (c) 5% Resin 95% Aluminum
Roughness profile amplitude parameters—ISO4287			
Ra [µm]	3.85	3.06	0.572
Rz [µm]	20.9	21.8	4.31
Rp [µm]	10.1	8.27	1.48
Rv [µm]	10.8	13.6	2.83
Rsk	−0.373	−2.56	−31.4
Rku	3.22	15.2	562
Roughness profile height parameters—ISO25178			
Sq [µm]	7.17	7.95	2.22
Sp [µm]	25.6	32.9	19.5
Sv [µm]	46.8	94.4	62.4
Sz [µm]	72.4	127	81.9
Sa [µm]	5.56	4.36	0.907
Ssk	−0.808	−3.16	−8.47
Sku	4.16	18.2	150
Surface functional parameters—ISO25178			
Rmr [%]	0.14	0.307	0.958
Smq [%]	83.5	92.3	98.3
Svq [µm]	12.6	16.4	24.0
Spq [µm]	5.25	2.25	0.977

## Data Availability

Data are contained within the article.
